# Chronic myeloid leukemia with an e1a3 BCR-ABL fusion protein: transformation to lymphoid blast crisis

**DOI:** 10.1186/2050-7771-2-14

**Published:** 2014-08-14

**Authors:** Jordi Martinez-Serra, Raquel del Campo, Antonio Gutierrez, Jose Luis Antich, Magdalena Ginard, Maria A Durán, Leyre Bento, Teresa Ros, Juan C Amat, Carmen Vidal, Julio F Iglesias, Izabela Orlinska, Joan Besalduch

**Affiliations:** 1 Department of Hematology, University Hospital Son Espases, Palma de Mallorca, Spain; 2 Department of Hematology, Hospital Son Llatzer, Palma de Mallorca, Spain; 3 Department of Hematology, Clinica Rotger, Palma de Mallorca, Spain; 4 Core of Sequentiation, University Hospital Son Espases, Palma de Mallorca, Spain; 5 Department of Immunology, University Hospital Son Espases, Palma de Mallorca, Spain; 6 Instituto de Investigación Sanitaria de Palma (IdISPa), Palma de Mallorca, Spain

**Keywords:** bcr-abl, e1a3, CML, ALL

## Abstract

Chronic myelogenous leukemia (CML) results from the neoplastic transformation of a hematopoietic stem cell. CML is cytogenetically characterized by the presence of the Philadelphia chromosome (Ph’). Most patients with CML express e13a2 or e14a2 mRNAs that result from a rearrangement of the major breakpoint cluster regions (M-*BCR*) generating the 210-kDa (p210BCR-ABL) fusion proteins b2a2 or b3a2 respectively. The e1a3 CML-related atypical translocation has been reported with an indolent clinical course, low leukocyte count, long chronic phase even without treatment and good response to therapy. We report the case of a patient initially diagnosed as CML in chronic phase whose cells expressed the e1a3 variant. The patient readily responded to imatinib 400 mg with the achievement of a rapid complete cytogenetic response and the normalization of the blood count values, but after 5 months transformed into lymphoid blast crisis.

## Background

Chronic myelogenous leukemia (CML) results from the neoplastic transformation of a hematopoietic stem cell. This leukemia is cytogenetically characterized by the presence of the Philadelphia chromosome (Ph’), which results from the reciprocal translocation t(9;22) (q34;q11) that juxtaposes the c-abl oncogene 1 (ABL1) gene on chromosome 9 with the breakpoint cluster region (BCR) gene on chromosome 22 generating the BCR-ABL1 oncogene [1-3]. The BCR-ABL fusion protein is the product of the Philadelphia chromosome [4]. Depending on the location of the breakpoint in BCR, several types of BCR-ABL fusion protein may be formed [5]. To date 3 main breakpoint cluster regions in the BCR gene have been reported: The M-bcr region located between exons 12 and 16, the m-bcr located between exons e2′ and e2 and the u-bcr located in exon 19 [5-7]. The point of rupture in the ABL gene usually occurs in exon 2 (a2). Most patients with CML express e13a2 or e14a2 mRNAs that result from a rearrangement of the major breakpoint cluster regions (M-*BCR*) generating the 210-kDa (p210BCR-ABL) fusion proteins b2a2 or b3a2 respectively, mainly associated to CML. Another typical breakpoint within the BCR occurs in exons 1 (e1) and 19 (e19) generating the rearrangements e1a2 (p190BCR-ABL) or e19a2 (p230BCR-ABL), associated to acute lymphoblastic leukemia (ALL) or neutrophil CML, respectively [5-8]. The type of rearrangement in CML is thought to be related to the patient clinical course. Here we report a CML case with the rare e1a3 translocation. One of the main features of this type of translocation is the absence of the exon a2, normally present in the other translocations. The e1a3 BCR-ABL1 related CML has been reported with an indolent clinical course, low leukocyte count, long chronic phase even without treatment and good response to therapy [9,10]. The abl exon a2 sequence, code for a part of the SH3 region of the abl protein, involved in the negative regulation of the kinase domain. *Bcr-Abl* mutants with deleted SH3 induce growth-factor independence and transform murine bone marrow, but leukemic cell proliferation in vivo is delayed as a result of reduced tissue invasiveness or leukemogenesis [9,11,12]. We report the case of a patient initially diagnosed as CML in chronic phase whose cells expressed the e1a3 variant. This patient readily responded to imatinib 400 mg with the achievement of a rapid complete cytogenetic response and the normalization of the blood count values, but after 5 months transformed into lymphoid blast crisis.

## Case presentation

**Figure 1:**
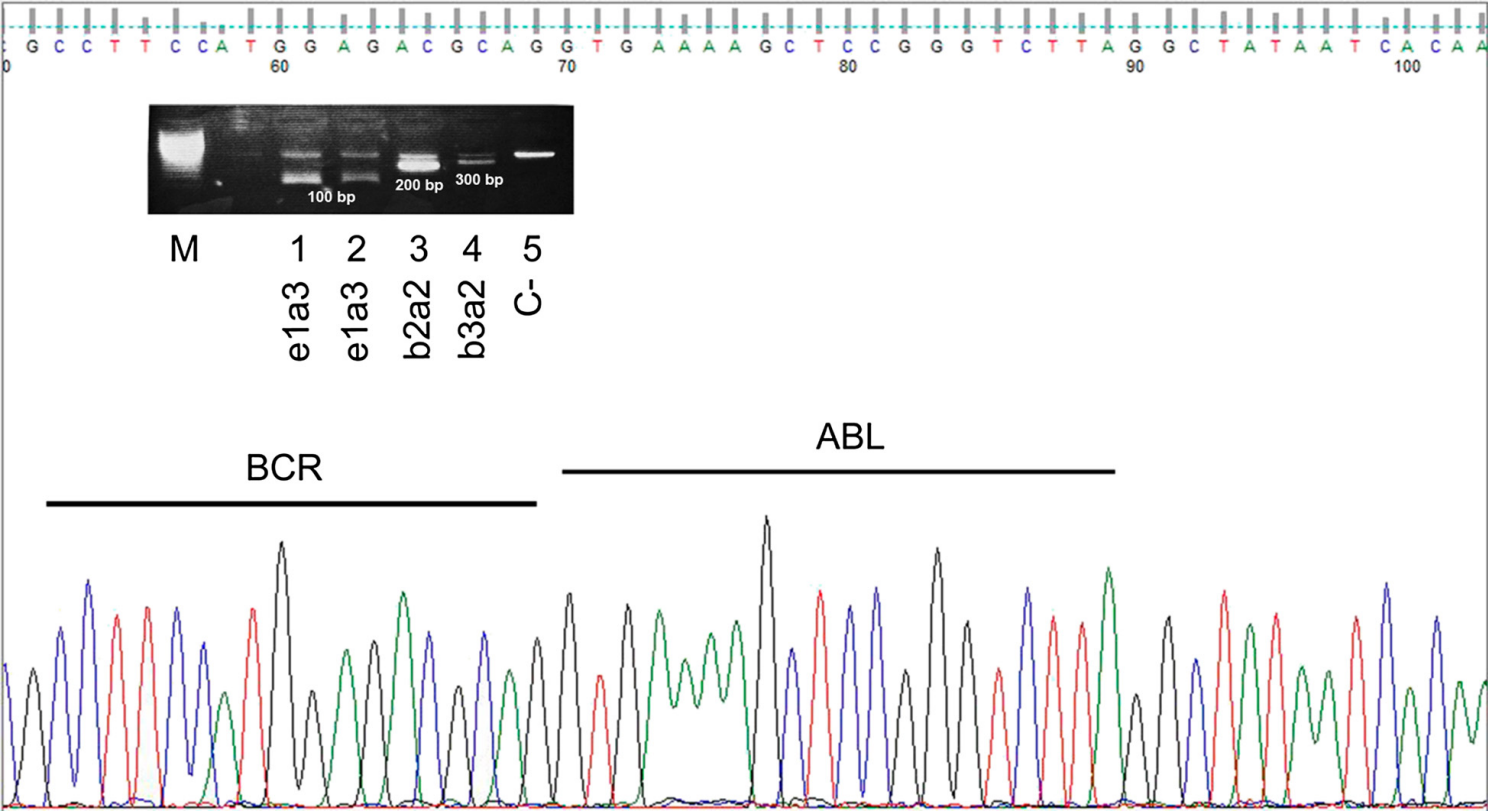
**DNA Sequencing of BCR-ABL e1a3 atypical translocation.** We observed an amplification band of approximately 100 bp (e1a3). Besides this band we included the b2a2 (200 bp) and b3a2 (300 bp) BCR-ABL control bands. In order to confirm the presence of a BCR-ABL transcript this band was extracted from the agarose gel, purified and then analyzed by DNA sequencing.

**Figure 2:**
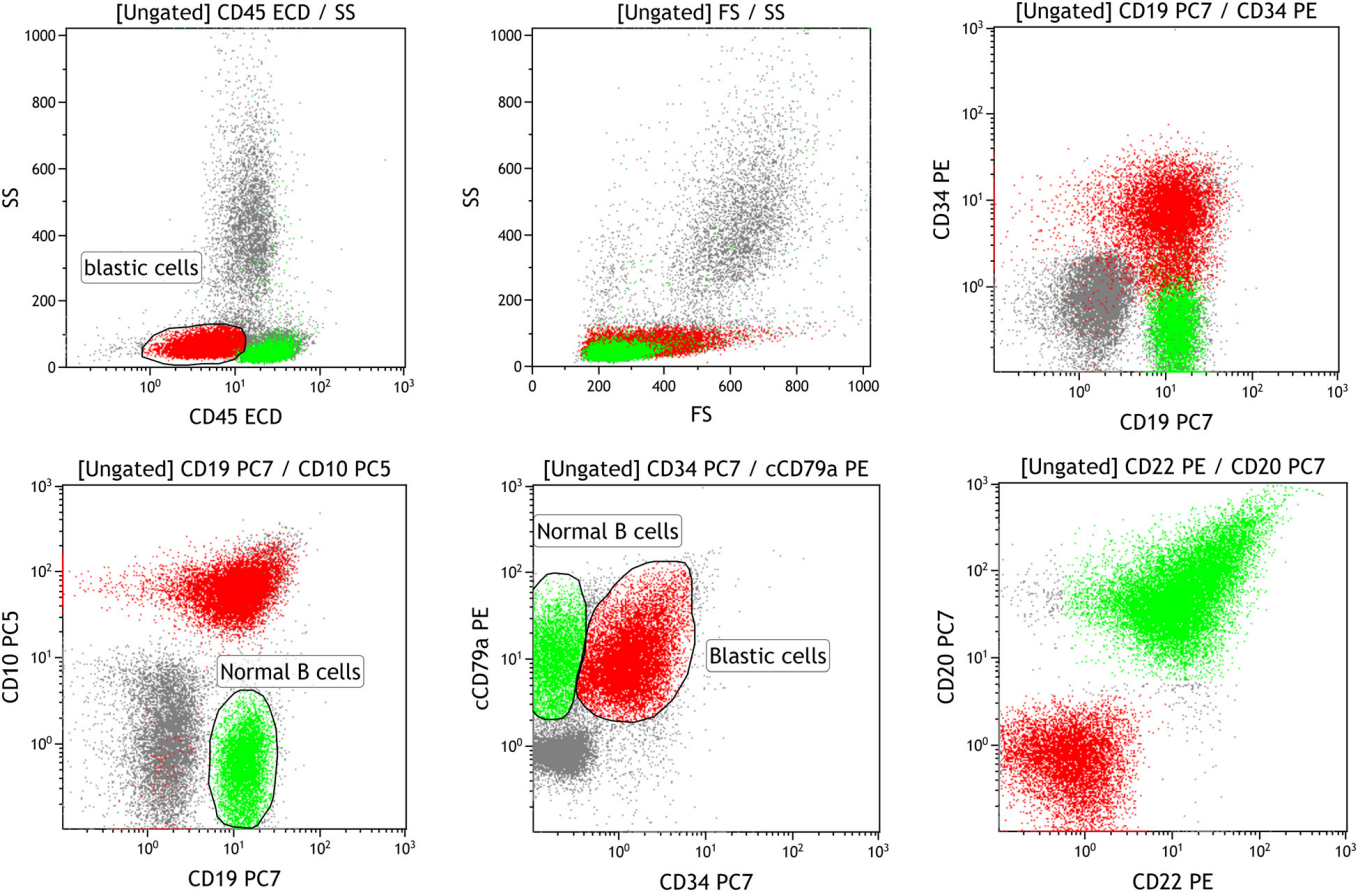
**Immnunophenotype of ALL with atypical e1a3 translocation.** Presence of a 70% of blasts with lymphoblastic phenotype B: CD19+, CD10+, CD34+, DR+, CD20 - CD22 -, cytoplasmic IgM negative and positive alpha CD79.

An 80-year-old Caucasian male sought medical attention due to 2 months of progressive asthenia, shortness of the breath, fatigue, and weight loss. The peripheral blood findings were mild anaemia, haemoglobin 11.8 g/dl, mild leukocytosis 21 × 10^9^/L with myelocytes 17%, metamyelocytes 6% and normal platelets. The bone marrow aspirate examination was hypercellular, with bone smear showing no myeloblasts evidence (or <1% myeloblasts) and 90% of myeloid cells. A fresh sample from the bone marrow aspirate was collected for cytogenetic and FISH analysis. They revealed the presence of a karyotype 46XY, t(9;22) (q34;q11.2) that confirmed the diagnosis of CML in chronic phase. We highlight that no additional chromosomal alterations were revealed even after the evolution to blast crisis. The FISH study was positive for the BCR/ABL in 50% of interphase cells analyzed. The patient’s RNA was isolated from the peripheral blood and subjected to a two round multiplex RT-PCR reaction. In order to avoid RNA quality and/or handling errors, we included an internal positive control in which a 690-bp segment of the ubiquitously expressed transcription factor E2A mRNA was amplified. The primers and PCR conditions used in the first and second round of the nested PCR reaction are described by Pallisgaard et al. [13]. We identified an atypical amplification band of approximately 100 bp. In order to confirm the presence of a BCR-ABL transcript this band was extracted from the agarose gel, purified and then analyzed by DNA sequencing. cDNA sequence confirmed the presence of the e1a3 BCR-ABL transcript (Figure 1). The initial management with 400 mg/day of Imatinib readily led to an improvement of clinical and analytical signs with complete normalization of blood counts. After 3 months a complete BM cytogenetic remission was achieved. The myelogram showed myeloblasts 2%, promyelocytes 5%, myelocytes 12%, metamyelocytes 36%, eosinophils 5%, lymphocytes 7% and monocytes 1%. BM smears were normocellular with discrete erythroid hypoplasia and no basophilia was observed. Immunophenotype studies showed no abnormal cell populations. After five months of follow up, the patient presented with a lymphoid blast crisis with 94% of blasts. In BCR-ABL (e1a3) positive cells cytogenetic analysis revealed no additional abnormalities apart from the presence of the Ph- Chromosome. Immunophenotype of the blast cells was as follows: Presence of 70% of blasts with lymphoblastic phenotype B: CD19+, CD10+, CD34+, DR+, CD20 - CD22 -, cytoplasmic IgM negative and positive alpha CD79. The rest of myeloid and T-cell studied markers expression were negative (Figure 2).

## Discussion

The rare e1a3 translocation has already been reported in the literature in 3 patients with CML in chronic phase and in 10 patients with ALL [9,10,13-17]. Surprisingly, most previously reported cases were detected in ALL patients and only in a few patients with CML in chronic phase. This fact together with the benign or indolent course of the CML with low leukocyte counts could lead to an underdiagnosed malignancy [9]. Moreover, the difficulties in detecting this atypical translocation with platforms such as GeneXpert could enhance the diagnostic problems of this type of translocation [9]. We hypothesize that these e1a3 ALL could represent lymphoid blast crisis of underdiagnosed e1a3 CML. To our knowledge there are no myeloid blast crisis described with the e1a3 rearrangement. Deletion of the exon a2 (ABL) from the BCR-ABL fusion transcript results in a protein that lacks the N-terminal two thirds of the *Src* homology 3 (SH3) domain [9,16]. Several authors have already indicated that the SH3 domain of the chimeric tyrosine kinase is not necessary for the activation of intracellular signals regulating proliferation and survival (RAS, PI-3K, JNK, MAPK, STATs and c-MYC) of hematopoietic cells but it is essential for full leukemogenic potential in vivo [11,12]. In this context, and based on a few cases, the presence of this translocation has been associated with good outcome of the disease for CML [9]; there is no such evidence for ALL. However due to the reduced number of e1a3 CML cases, it is necessary to accumulate more clinical evidences in order to clarify the relationship between the presence of the e1a3 BCR-ABL translocation and their clinical course that could be similar or worse than standard p210 CML as it could confer a higher risk of transformation to ALL.

## Conclusion

This report describes the first case of a CML in chronic phase with an e1a3 translocation (with a suggested indolent course), which after 5 months in complete cytogenetic remission transformed into a lymphoid blast crisis. This case suggests that although imatinib therapy is able of inducing a very favorable response in the patient does not exempt them from undergoing to blast crisis. On the other hand, we provide a rationale to consider that e1a3 CML may have a tendency to progress to a lymphoid blast crisis, sometimes with the chronic phase underdiagnosed.

## Consent

Written informed consent was obtained from the patient’s next of kin for publication of this case report and any accompanying images. A copy of the written consent is available for review by the Editor-in-Chief of this journal.

## Competing interests

The authors declare that they have no competing interests.

## Authors’ contributions

JM-S, AG and JB, reviewed the literature and wrote the paper. RC and JLA: treated the patient and collected the data. JMS, MG, JCA TR, LB, JI, MAD, IO and CV, performed the molecular/immune analysis. All authors carried out critical interpretations, read and approved the final manuscript.
